# Multiple injections of human umbilical cord-derived mesenchymal stromal cells through the tail vein improve microcirculation and the microenvironment in a rat model of radiation myelopathy

**DOI:** 10.1186/s12967-014-0246-6

**Published:** 2014-09-08

**Authors:** Li Wei, Jing Zhang, Xiu-Bin Xiao, Hai-Xing Mai, Ke Zheng, Wan-Liang Sun, Lei Wang, Feng Liang, Zai-Liang Yang, Yuan Liu, Yan-Qing Wang, Zhi-Fang Li, Jia-Ning Wang, Wei-Jing Zhang, Hua You

**Affiliations:** Affiliated Hospital of the Academy of Military Medical Sciences, No.8 East Main Street, Fengtai District, Beijing, 100071 China; Key Laboratory of Birth Defects and Reproductive Health of the National Health and Family Planning Commission, Chongqing Population and the Family Planning Science and Technology Research Institute, Chongqing, 400020 China; Department of Endocrine Surgery, the First Affiliated Hospital of Chongqing Medical University, Chongqing, 400016 China; Department of Cardiology & Institute of Clinical Medicine, Hubei University of Medicine, Shiyan, Hubei 442000 China; Research Institute of Surgery, Daping Hospital, Third Military Medical University, Chongqing, 400042 China; The First Affiliated Hospital of Liaoning Medical University, No.2 Renmin Street, Guta District, Jinzhou, 121000 Liaoning Province China; PLA 205 Hospital, No.9 Chongqing Road, Guta District, Jinzhou, 121000 Liaoning Province China

**Keywords:** Radiation myelopathy, Endothelial cell, Spinal cord blood flow, Paracrine system, Human umbilical cord-derived mesenchymal stromal cells

## Abstract

**Background:**

At present, no effective clinical treatment is available for the late effects of radiation myelopathy. The aim of the present study was to assess the therapeutic effects of human umbilical cord-derived mesenchymal stromal cells (UC-MSCs) in a rat model of radiation myelopathy.

**Methods:**

An irradiated cervical spinal cord rat model was generated. UC-MSCs were injected through the tail vein at 90, 97, 104 and 111 days post-irradiation. Behavioral tests were performed using the forelimb paralysis scoring system, and histological damage was examined using Nissl staining. The microcirculation in the spinal cord was assessed using von Willebrand factor (vWF) immunohistochemical analysis and laser-Doppler flowmetry. The microenvironment in the spinal cord was determined by measuring the pro-inflammatory cytokines interleukin-1β (IL-1β) and tumor necrosis factor-α (TNF-α) in the serum and the anti-inflammatory cytokines brain-derived neurotrophic factor (BDNF) and glial cell-derived neurotrophic factor (GDNF) in the spinal cord.

**Results:**

Multiple injections of UC-MSCs through the tail veil decreased the forelimb paralysis, decreased spinal cord histological damage, increased the number of neurons in the anterior horn of the spinal cord, increased the endothelial cell density and the microvessel density in the white matter and gray matter of the spinal cord, increased the relative magnitude of spinal cord blood flow, down-regulated pro-inflammatory cytokine expression in the serum, and increased anti-inflammatory cytokine expression in the spinal cord.

**Conclusion:**

Multiple injections of UC-MSCs via the tail vein in a rat model of radiation myelopathy significantly improved the microcirculation and microenvironment through therapeutic paracrine effects.

## Introduction

The spinal cord is a well-known example of “late-reacting” tissue in response to irradiation [1]. Exposure of the spinal cord to radiation can result in radiation myelopathy, which is a rare but serious complication of radiotherapy for cancer [2]. The late effects of radiation myelopathy (i.e., those occurring 6 months to several years after treatment) can be extremely severe and may seriously decrease the patient’s quality of life. These effects are particularly dangerous because they are generally irreversible [3,4]. Currently, there is no effective clinical treatment for the late effects of radiation myelopathy.

Two distinct hypotheses—the glial hypothesis (demyelination) and the vascular hypothesis—have been developed to explain the underlying mechanisms leading to the development of selective white matter necrosis and, in particular, to identify the primary “target cell population” within the CNS [4]. Vascular damage is considered the key step in the development of radiation myelopathy [1,2,4–8]. Hence, the vascular endothelial cell population has been proposed as one of the most critical targets of the late effects of radiation myelopathy [9].

Previous studies have examined early endothelial cell apoptosis in the spinal cord after irradiation [10,11]. Radiation-induced apoptosis of endothelial cells has been shown to depend on the ASMase pathway rather than the p53 pathway [10]. Because we have clearly determined the time course of the endothelial cell response within 180 days post-irradiation and demonstrated its relationship with spinal cord blood flow [12], it seems possible that targeting endothelial cell death may be a neuroprotective strategy that can limit the late effects of spinal cord irradiation [12,13].

Recently, multipotent mesenchymal stromal cell (MSC) treatment has attracted special attention as a new alternative strategy for stimulating regeneration. MSCs are primitive cells originating from the mesodermal germ layer and are classically described as giving rise to connective tissues, skeletal muscle cells, and cells of the vascular system [14]. MSCs may exhibit immunosuppressive properties and have been suggested to be “immune-privileged”. They are thus protected from rejection, potentially permitting their use in allo-transplantation. It appears that MSCs have the capacity to localize to injured tissue and differentiate into specific cell types [15]. Some reports suggest that the therapeutic effects of MSCs in injured tissue are mainly mediated by paracrine activity, including the stimulation of endogenous repair, angiogenesis and arteriogenesis; attenuation of remodeling; and reduction of apoptosis [16]. Interestingly, injected murine MSCs, but not human MSCs, differentiated into osteosarcomas in injured lungs. Thus, human MSCs appear to be more feasible and safer for use than murine MSCs [17].

The aim of the present study was to assess the therapeutic effects of human umbilical cord-derived mesenchymal stromal cells (UC-MSCs) in a rat model of radiation myelopathy. To the best of our knowledge, this is the first clinically based translational study to assess and highlight human MSC therapy for the treatment of radiation myelopathy.

## Materials and methods

### Animals

Adult female Sprague–Dawley (SD) rats (160–200 g, Laboratory Animal Center, Academy of Military Medical Sciences, Beijing, China) were housed and cared for according to the guidelines for the care and use of laboratory animals of the NIH and Academy of Military Medical Sciences (Beijing, China). All experimental procedures were approved by the Committee for Animal Use at the Academy of Military Medical Sciences. Every effort was made to minimize the number of animals used as well as their suffering. Water and food were available ad libitum in the cages.

### Isolation and differentiation of human UC-MSCs

UC-MSCs were isolated from Wharton’s jelly (WJ) of umbilical cords according to previously described methods with some modification [18]. Fresh umbilical cords were collected after obtaining consent from the mothers. The umbilical cords were rinsed in phosphate-buffered saline (PBS) until the cord blood was cleared, and the blood vessels were removed. The remaining WJ tissue was cut into 1-2 mm^3^ pieces and placed in six-well plates in the presence of 0.1% collagenase type II (Sigma, USA) in PBS at 37°C for 1 h. Ten percent fetal bovine serum (FBS, Invitrogen, USA) was then added to stop the digestion. The dissociated mesenchymal cells were dispersed in 10% FBS-DMEM and further cultured until well-developed colonies of the fibroblast-like cells reached 80% confluence. Then, the cultures were trypsinized with 0.25% trypsin-EDTA (Invitrogen, USA) and passaged into new flasks for further expansion. The multipotent differentiation capacity of the UC-MSCs was confirmed by their differentiation into adipocytes, chondroblasts and osteoblasts using Oil Red O staining (adipocytes), alcian blue staining (chondroblasts), and alkaline phosphatase (osteoblasts), respectively. The surface markers of the UC-MSCs were also examined by flow cytometry.

### Irradiation and UC-MSC injection

A total of 20 SD rats were randomly divided into 4 groups (n = 5 each): normal control (control) group, untreated irradiation (irradiation) group, PBS treatment (PBS) group, and UC-MSC treatment (UC-MSC) group. Each rat in the irradiation, PBS and UC- MSC groups was anesthetized using 3% sodium pentobarbiturate (45 mg/kg) delivered by intraperitoneal injection and subsequently irradiated using a ^60^Co source. A ^60^Co irradiator (Model GWXJ80, NPIC, Chengdu, China) was used to conduct gamma ray irradiation, and the rats were irradiated with 30 Gy at a dose rate of approximately 150 cGy/min. The beam was strictly limited to a 2-cm segment of the cervical spine field spanning C2-T2. According to previous reports [2,10,11,19], forelimb paralysis associated with white matter necrosis occurs approximately 20 weeks following the delivery of single doses of 20 Gy or more to the cervical spinal cord in this radiation myelopathy model. Single doses of 19.5 and 22 Gy represent the ED50 and ED100, respectively, for forelimb paralysis within 180 days secondary to white matter necrosis in the rat spinal cord model. Rats in the PBS and UC-MSC groups were given PBS and UC-MSC (1 × 10^6^ cells) injections through the tail vein in a 200 μl volume at 90, 97, 104 and 111 days post-irradiation. Rats in the irradiation group were not provided any treatment after irradiation. Rats in the control group were fed normally and not irradiated during the same period. The rats were observed daily for up to 180 days post-irradiation.

### Assessment of motor function

At 180 days post-irradiation, the motor function of five rats was examined using a slightly modified version of the methodology described by Chiang [20]. A technical assistant who was blinded to the experimental conditions evaluated the rats’ motor function. In the subjective scoring system that was used, 0 represented total paralysis of both forelimbs, 1 represented definite sub-total paralysis (one forelimb paralyzed or slight movement of both forelimbs; toes do not spread when the animal is held up), 2 represented definite abnormal movement (very unsteady movements, low stance, uncoordinated movements, hops), 3 represented questionably abnormal movement (slightly wobbly or uncoordinated movements), and 4 represented normal movement.

### Assessment of spinal cord blood flow (SCBF)

SCBF was measured using a MoorLab laser-Doppler flowmeter (MoorLab Instruments, Devon, England) as described in our previous study [12]. This instrument recorded the relative change in blood flow over time. Briefly, the animals were mounted on a stereotactic instrument equipped with a vertebral fixation device to stabilize the vertebral column. Body temperature was maintained at 37.0 ± 0.5°C using a heating blanket. The laser-Doppler probe was affixed to a micromanipulator and placed perpendicular to the spinal cord, barely touching the dorsal surface of the dura mater. Laser-Doppler signal readings were recorded on a computer and were analyzed using Moorsoft for Windows (version 1.31). SCBF was measured before irradiation (0 d) and 180 d after irradiation. The mean value before irradiation (0 h) was used as the basal value, and the final value for each rat at each later time point was expressed as a percentage of the basal value (100%).

### Specimen processing and histology

After the SCBF assessment at 180 days post-irradiation, blood samples were collected from the heart and allowed to clot for 2 h at room temperature. Serum was obtained after centrifugation at 2000 rpm for 20 min at 4°C and stored at −80°C. The C2-T2 spinal cord segment was removed, and a 1-cm-long central section of this segment was cut and immersed in 4% paraformaldehyde for histological and immunohistochemical analysis. The remaining portion of the spinal cord was frozen in dry ice power and stored at −80°C prior to use. The previously mentioned 1-cm-long central section of the C2-T2 spinal cord segment was dehydrated in ethanol and embedded in paraffin. The sections (5 μm thickness) were deparaffinized, rehydrated, and stained using Nissl staining. For Nissl staining, the sections were incubated with 0.1% cresyl violet at 37°C for 10 min and subsequently dehydrated, mounted, and observed under a light microscope. Neurons with diameters > 20 μm in both anterior horns of the spinal cord were counted.

### Immunohistochemical analysis

The sections (5 μm thickness) were subjected to microwave antigen retrieval using Tris-EDTA (pH 9, 10 min). Endogenous peroxidase was quenched using 3% H_2_O_2_ for 10 min. The sections were incubated with polyclonal rabbit anti-human von Willebrand factor (vWF, also called factor VIII-related antigen) antibody (1:100 dilution; DakoCytomation, Denmark) or anti-human nuclei antibody (MAB1281, 1:1000 dilution; Millipore) at 37°C for 3 h. The primary antibody was omitted from the negative control. After washing with PBS, the sections were incubated with biotinylated monoclonal anti-rabbit IgG for 20 min at room temperature using a SABC Detection System (Boster Biological Technology, Wuhan, China) according to the manufacturer’s protocol. The slides were developed with diaminobenzidine (DAB; DakoCytomation, Denmark), counterstained with Mayer’s hematoxylin, dehydrated with increasing concentrations of alcohol, cleared in xylene, and mounted in neutral balsam (Sigma, USA).

### ELISA

The levels of interleukin-1β (IL-1β) and tumor necrosis factor-α (TNF-α) in the serum and the levels of brain-derived neurotrophic factor (BDNF) and glial cell-derived neurotrophic factor (GDNF) in the spinal cord tissues were determined using commercially available ELISA kits according to the manufacturer’s protocols (IL-1β and TNF-α, Invitrogen, Carlsbad, California, USA; BDNF, MyBioSource, SanDiego, California, USA; GDNF, Abnova, Taipei city, Taiwan). The OD value was determined by an ELISA reader at a wavelength of 450 nm and calculated in the linear part of the curve.

### Morphometric analysis

For the Nissl staining, two sections per rat were selected in every sixth section, and a total of ten sections per time point were carefully examined at 100X magnification under an Olympus microscope [12]. According to the methodology described previously by Li et al. and our group [10–12], the endothelial cell density was defined as the total number of endothelial cells that contained a nucleus divided by the transverse cross-sectional area of the spinal cord, and the microvessel density was defined as the total number of microvessels that were transversely sectioned and contained either an endothelial cell nucleus or no nucleus divided by the transverse cross-sectional area of the spinal cord. One hotspot area in the gray matter and another hotspot area in the white matter were selected at 100X magnification. The densities of the endothelial cells and microvessels were then determined using two fixed fields (0.768 mm^2^) within each of these two areas at 400X magnification per section. Three sections per rat were selected in every sixth section, and a total of thirty fields per time point in the gray matter and thirty fields per time point in the white matter were used for the analysis. Image-ProPlus software was used for the analyses. For morphometric analysis, two blinded pathologists carefully and independently examined all selected sections. The number of neurons, the endothelial cell density and the microvessel density were determined in the individual analyses, and mean values were calculated. We used some data from our previous paper [12] as the same experimental conditions which were employed in the present work.

### Statistics

All quantitative data are expressed as the mean value ± SEM. Differences were evaluated using one-way ANOVA. Comparisons between values at two time points were performed using the least significant difference (LSD) procedure. A *P* value *<* 0.05 was considered statistically significant.

## Results

### Characterization of UC-MSCs

**Figure 1:**
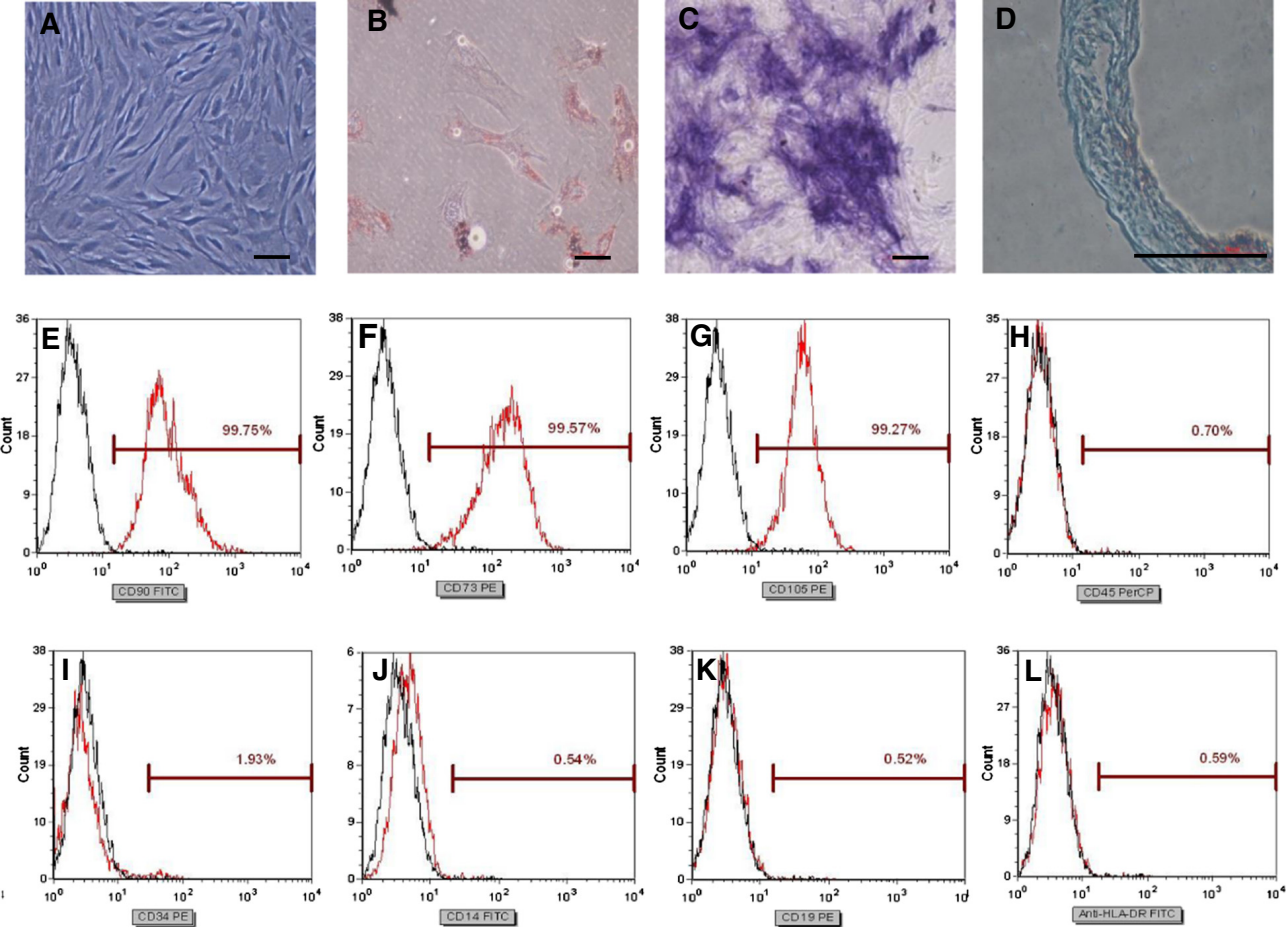
**Culture and identification of UC-MSCs. (A)** UC-MSCs exhibiteda spindle- and fibroblast-like shape. **(B-D)** Multipotential differentiation of UC-MSCs. UC-MSC differentiation into adipocytes, osteoblasts and chondroblasts, as shown by Oil Red O **(B)**, alkaline phosphatase **(C)**, and alcian blue **(D)** staining, respectively, of in vitro differentiation cultures. **(E-L)** Flow cytometry analysis of surface markers inUC-MSCs. Most of the cultured cells were positive for expression of CD90 **(E)**, CD73 **(F)** and CD105 **(G)** but were negative for expression of CD45 **(H)**, CD34 **(I)**, CD14 **(J)**, CD19 **(K)** and HLA-DR **(L)**. The scale bar is 10 μm in A, 100 μm in **B** and **C**, and 50 μm in **D**.

The UC-MSCs exhibited similar spindle- and fibroblast-like shapes (Figure 1A). The multipotent differentiation capacity of the UC-MSCs was confirmed by their differentiation into adipocytes, osteoblasts and chondroblasts, as shown by the staining of the in vitro differentiation cultures with Oil Red O (Figure 1B, adipocytes), alkaline phosphatase (Figure 1C, osteoblasts), and alcian blue (Figure 1D, chondroblasts). The surface markers of the UC-MSCs were examined by flow cytometry. Most of the cultured cells were positive for CD90, CD73 and CD105 expression but negative for CD45, CD34, CD14, CD19 and HLA-DR expression (Figure 1E-L).

### UC-MSC administration improved locomotion recovery

**Figure 2:**
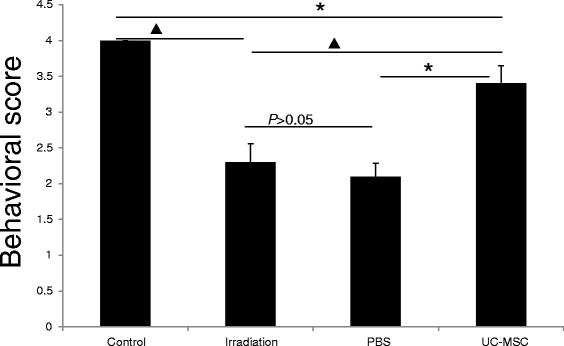
UC-MSC administration improved locomotion recovery at 180 days post-irradiation (n = 5, * ***P*** **< 0.05,** ^**▲**^ ***P*** **< 0.01).** Error bars represent the S.E.M.

To determine the potential benefit of the UC-MSC infusion on the spinal cord after irradiation, behavioral tests were performed using the forelimb paralysis scoring system. Although we did not observe total paralysis at 180 days post-irradiation in our radiation myelopathy rat model, in which demyelination did not occur, the irradiated rats still exhibited significant forelimb motor function impairment compared with the normal controls at 180 days post-irradiation [12]. In irradiated animals, UC-MSC treatment significantly decreased the forelimb paralysis, compared with PBS treatment and no treatment, indicating that irradiated animals treated with UC-MSCs exhibited improved motor function (indicated by a higher score on the test). There were no significant differences in the forelimb paralysis score between PBS-treated irradiated animals and untreated irradiated animals (Figure 2).

### UC-MSC administration ameliorated irradiation-induced histological damage in the spinal cord

**Figure 3:**
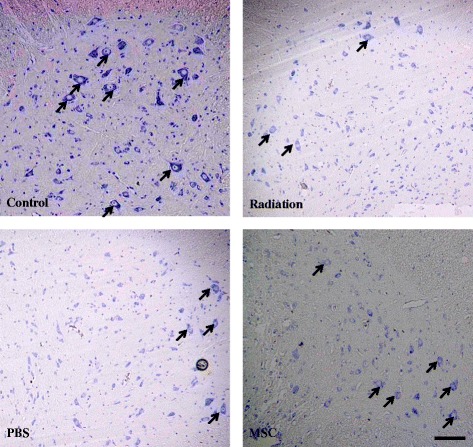
**UC-MSC administration decreased irradiation-induced histological damage in the spinal cord.** Representative micrographs of Nissl-stained rat spinal cords at 180 days post-irradiationfollowing systemic injection of UC-MSCs. Arrows indicate Nissl bodies. Scale bars = 100 μm.

Next, we evaluated whether UC-MSC injection could decrease histological damage to the spinal cord after irradiation. We previously determined that a single 30-Gy dose of irradiation primarily injured the neurons in the spinal cord. A markedly thickened cytoplasm, significant swelling and distension of the soma, and hazy Nissl bodies were observed in the neurons between 30 days and 90 days post-irradiation. After 90 days post-irradiation, this neuronal damage was reversed to some degree [12]. Histological examination of spinal cord sections using Nissl staining revealed neuronal damage similar to that observed in the untreated irradiated animals and in the PBS-treated animals at 180 days post-irradiation (Figure 3). Interestingly, the neurons of UC-MSC-treated irradiated animals were less damaged than those of untreated irradiated animals (Figure 3).

**Figure 4:**
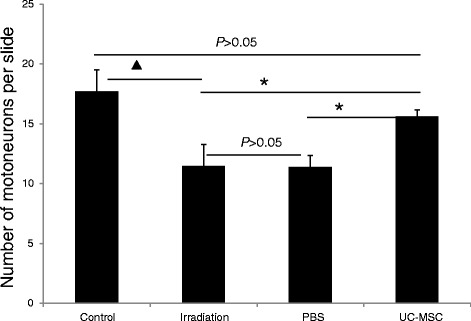
UC-MSC administration improved neuron survival in the anterior horn at 180 days post-irradiation (n = 10, * ***P*** **< 0.05,** ^**▲**^ ***P*** **< 0.01).** Error bars represent the S.E.M.

We also counted the number of neurons in the anterior horn of the spinal cord following irradiation using Nissl staining. The number of neurons in the UC-MSC-treated animals (15.6 ± 0.6) was significantly higher than that in the PBS-treated animals (11.4 ± 1.0) and that in the untreated irradiated animals (11.5 ± 1.8) at 180 days post-irradiation (Figure 4). The number of neurons in the PBS-treated animals was similar to that in the untreated irradiated animals (Figure 4).

### UC-MSC administration improved irradiation-induced microcirculation dysfunction in the spinal cord

**Figure 5:**
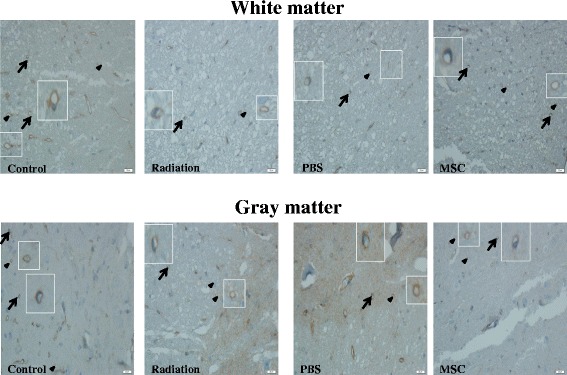
**Representative micrographs of von Willebrand factor immunohistochemical staining of the rat spinal cord after a single dose of 30 Gy.** Arrows,endothelial cells; arrowheads,microvessels. Scale bars = 20 μm.

**Figure 6:**
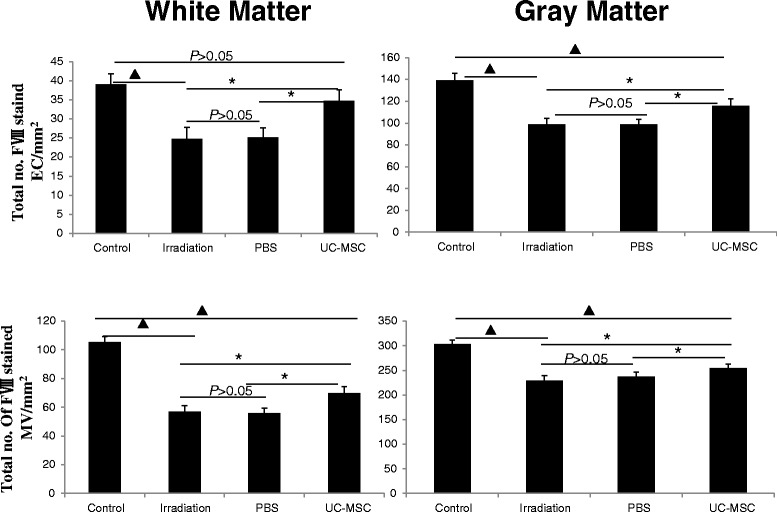
UC-MSC administration increased the endothelial cell density (n = 30) and microvessel density (n = 30) in the spinal cord at 180 days post-irradiation (* ***P*** **< 0.05,** ^**▲**^ ***P*** **< 0.01).** Error bars represent the S.E.M.

To explore angiogenesis induced by UC-MSC administration, microvessels were identified with anti-factor VIII antibody, a specific marker for endothelial cells. The endothelial cell density and microvessel density were then quantified in the white matter and gray matter of the spinal cord (Figure 5). The endothelial cell and microvessel densities were significantly higher in the UC-MSC-treated animals [endothelial cell density: (34.7 ± 2.8)/mm^2^ in the white matter, (115.9 ± 6.2)/mm^2^ in the gray matter; microvessel density:(69.9 ± 4.6)/mm^2^in the white matter, (255.2 ± 7.2)/mm^2^ in the gray matter] than those in the PBS-treated animals [endothelial cell density: (25.2 ± 2.5)/mm^2^ in the white matter, (99.0 ± 4.6)/mm^2^ in the gray matter; microvessel density: (56.0 ± 3.5)/mm^2^ in the white matter, (237.4 ± 9.1)/mm^2^ in the gray matter] and those in the untreated irradiated animals [endothelial cell density (24.7 ± 3.0)/mm^2^ in the white matter, (99.0 ± 5.3)/mm^2^ in the gray matter; microvessel density (56.9 ± 4.5)/mm^2^ in the white matter, (229.6 ± 9.6)/mm^2^ in the gray matter] at 180 days post-irradiation (Figure 6). The endothelial cell and microvessel densities were similar in the PBS-treated animals and the untreated irradiated animals (Figure 6). We also used a marker of human nuclei, MAB1281, to identify surviving UC-MSCs in spinal cord sections by immunohistochemistry. However, UC-MSCs were not found in spinal cord sections of UC-MSC-treated animals (data not shown).

**Figure 7:**
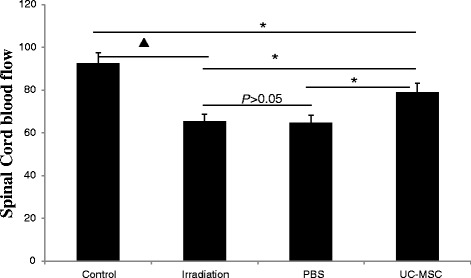
UC-MSC administration increased blood flow (n = 5) in the spinal cord at 180 days post-irradiation (* ***P*** **< 0.05,** ^**▲**^ ***P*** **< 0.01).** Error bars represent the S.E.M.

To address the functional effects of alterations in endothelialcell density and microvessel density, we applied laser-Doppler flowmetry to record the relative magnitude of the spinal cord blood flow [12]. The relative magnitude of the spinal cord blood flow in the UC-MSC-treated animals (79.0 ± 4.3) was significantly higher than that in the PBS-treated animals (64.8 ± 3.4) and untreated irradiated animals (65.5 ± 3.1) 180 days post-irradiation (Figure 7). The relative magnitude of the spinal cord blood flow in the PBS-treated animals and the untreated irradiated animals was similar (Figure 7).

### UC-MSC administration reversed irradiation-induced inflammation

**Figure 8:**
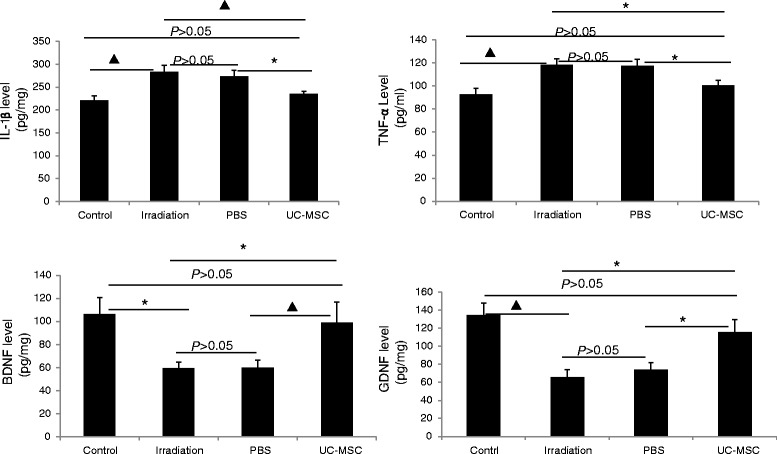
**UC-MSC administration reversed irradiation-induced inflammation.** Serum samples and spinal cord samples were collected from animals at 180 days post-irradiation following systemic injection of UC-MSCs and analyzed by ELISA (*P < 0.05, ^▲^ *P* < 0.01). Error bars represent the S.E.M.

We also determined whether the UC-MSC injection could reverse the spinal cord inflammation that arises after irradiation. We measured the levels of the pro-inflammatory cytokines IL-1β and TNF-α in the serum as well as the anti-inflammatory cytokines BDNF and GDNF in the spinal cord tissues to assess the inflammatory status. The irradiated rats exhibited significantly higher levels of IL-1β (283.2 ± 14.7 pg/ml) and TNF-α (118.2 ± 5.4 pg/ml) in the serum and lower levels of BDNF (59.7 ± 4.9 pg/mg) and GDNF (65.4 ± 8.4 pg/mg) in the spinal cord compared with the normal controls [IL-1β: 221.2 ± 9.8 pg/ml; TNF-α: 92.8 ± 5.0 pg/ml; BDNF:106.4 ± 14.1 pg/mg; GDNF:134.4 ± 13.6 pg/mg] at 180 days post-irradiation (Figure 8). The levels of IL-1β (235.4 ± 5.2 pg/ml) and TNF-α (100.5 ± 4.4 pg/ml) in the serum of the UC-MSC-treated animals were significantly lower than those in the PBS-treated animals [IL-1β: 273.8 ± 13.6 pg/ml; TNF-α:117.6 ± 5.3 pg/ml] and those in the untreated irradiated animals at 180 days post-irradiation (Figure 8). The levels of BDNF (99.0 ± 17.8 pg/mg) and GDNF (115.7 ± 14.2 pg/mg) in the spinal cord of the UC-MSC-treated animals were significantly higher than those in the PBS-treated animals [BDNF:60.1 ± 6.5 pg/mg; GDNF: 74.4 ± 7.5 pg/mg] and those in the untreated irradiated animals at 180 days post-irradiation (Figure 8). The levels of IL-1β and TNF-α in the serum and the levels of BDNF and GDNF in the spinal cord in the PBS-treated animals were similar to those in the untreated irradiated animals (Figure 8).

## Discussion

The primary aim of this work was to investigate the potential beneficial effects of the delivery of UC-MSCs through the tail vein on radiation myelopathy. In the present study, using a rat model of radiation myelopathy, we demonstrated that the administration of UC-MSCs through the tail vein improved motor function, as shown by decreased forelimb paralysis; decreased histological damage in the spinal cord, as shown by Nissl staining of spinal cord sections; increased the number of neurons in the anterior horn of the spinal cord, as shown by Nissl staining; increased endothelial cell density and the microvessel density in the white matter and gray matter of the spinal cord, as determined by vWF immunohistochemistry; increased relative magnitude of spinal cord blood flow, as measured by laser-Doppler flowmetry; down-regulated pro-inflammatory cytokine expression and increased anti-inflammatory cytokine expression, as measured by an analysis of IL-1β and TNF-α levels in the serum and BDNF and GDNF levels in the spinal cord. Our data not only define the potential beneficial roles of UC-MSC infusion through the tail vein but also provide the first evidence that UC-MSCs have paracrine therapeutic effects in a rat model of radiation myelopathy.

The beneficial effects of cell-based therapies have been demonstrated using different types of cells in pre-clinical models. One of the most extensively studied cell types used for traumatic spinal cord repair in experimental models with good potential for clinical translation are MSCs [21]. However, little is known about the mechanisms underlying the potential benefits of cell grafting into the injured spinal cord [22]. The beneficial effects of MSCs have been attributed to several potential mechanisms. MSCs produce neuroprotective molecules in injured tissue [23,24], promote vascularization [24], modulate the inflammatory response [22,25,26], and generate a permissive microenvironment for axonal regeneration [27–29]. In addition, MSCs have been shown to foster a positive relationship between the preservation of spinal cord parenchyma and functional and electrophysiological recovery [23,24,27], and they were shown to promote axonal regeneration when transplanted after complete spinal cordtransection [29]. The therapeutic effects of immortalized neural stem cells [2] and oligodendrocyte progenitor cells (OPCs) [30] were previously determined in a rat model of radiation myelopathy. The present study is the first to examine the beneficial effects of MSCs in an animal model of radiation myelopathy.

The therapeutic window for intervention may be a key factor that determines the beneficial effects of cell transplantation for radiation myelopathy. We applied laser-Doppler flowmetry to record the relative magnitude of the spinal cord blood flow and discovered that the lowest relative magnitude of spinal cord blood flow was reached at 90 days post-irradiation [12]. Therefore, we injected UC-MSCs at 90 days post-irradiation in our rat model of radiation myelopathy, using spinal cord blood flow as a reliable indicator of the optimal time for transplantation.

The transplantation route has also been suggested to be involved in the effectiveness of cell therapy [31]. For clinical application, intravenous injection appears to be the easiest method of delivering therapeutics to the patient without risking further damage to the spinal cord [24]. Further, the efficacy and feasibility of delivering MSCs by tail vein transfusion has been confirmed [24,32,33]. In our initial study, we injected UC-MSCs every 7 days through the tail vein and observed that the rats died if they received an injection more than 5 times (data not shown). Thus, we injected UC-MSCs through the tail vein at 4 time points: 90, 97, 104 and 111 days post-irradiation.

Compared to MSCs from other sources, the low immunogenicity and capacity for localized immunosuppression have been well documented for UC-MSCs [16]. UC-MSCs have been shown to survive, migrate over short distances, produce large amounts of BDNF and neurotrophin-3 (NT-3) in the host spinal cord, induce fewer reactive astrocytes, increase the length of neurofilament-positive fibers and the number of growth cone-like structures around the lesion site, and enhance hindlimb locomotor function [34]. Genetic modification of UC-MSCs with NT-3 further enhanced their therapeutic effectsafter clip injury to the spinal cord [35]. Injection of Schwann-like cells induced from UC-MSCs, combined with NT-3 administration, further promoted the survival of UC-MSCs and functional recovery [36]. A combination treatment of Taxol and UC-MSCs for spinal cord injury further reduced the extent of astrocyte activation, increased axonal preservation, decreased the number of caspase-3(+) and ED-1(+) cells, and enhanced functional recovery compared with UC-MSC treatment alone [37]. Our results demonstrated that UC-MSC administration decreased irradiation-induced histological damage in the spinal cord and ultimately improved locomotion recovery. These observations confirmed that the histological improvement induced by MSCs could result in a higher degree of functional recovery.

In the present study, UC-MSCs were not detected in spinal cord sections from UC-MSC-treated animals based on immunohistochemical staining of a marker of human nuclei, MAB1281. We did not exclude the possibility that a very limited number of UC-MSCs still survived in the radiation-injured spinal cord due to some limitations of immunohistochemical technology. Reports from the early period of MSC-based cell therapy for tissue repair demonstrated that injected MSCs may survive, engraft, differentiate into specific cell types, and repair injured tissues [15]. However, subsequent studies, including the present report, supported the notion that the level of engraftment of transplanted MSCs in the host organs of recipient animals was very low. The exact mechanisms of MSC action in tissue repair are poorly understood; however, the paracrine action of MSCs has been highlighted in previous papers [16]. Direct evidence for a paracrine role of MSCs has been obtained and confirmed in studies utilizing animal models of lung [38], heart [39], and liver [40] injury, which showed that conditioned medium (CM) generated from MSCs significantly reduced damage and stimulated regeneration in vivo. Down regulation of pro-inflammatory cytokines and up regulation of anti-inflammatory cytokines have been proposed as paracrine effects of MSCs [40]. Our results demonstrated that down regulation of the pro-inflammatory cytokines IL-1β and TNF-α in the serum occurred on a systemic level, and up regulation of the anti-inflammatory cytokines BDNF and GDNF in the spinal cord occurred on a local level. The controlled systemic and local inflammatory responses may contribute to endogenous regeneration mechanisms in the spinal cord.

MSCs have been demonstrated to increase the density of new blood vessels in traumatized spinal cord tissue, resulting in functional recovery [24,41]. We demonstrated that UC-MSC injection increased the endothelial cell density and the microvessel density in the white and gray matter of the spinal cord and increased spinal cord blood flow. This effect was induced by secreted factors that exert paracrine effects on local endothelial cells [42].

## Conclusions

In summary, we have demonstrated that multiple injections of UC-MSCs via the tail vein significantly improved the microcirculation and microenvironment through paracrine therapeutic effects in a rat model of radiation myelopathy. To the best of our knowledge, this is the first study to evaluate MSC therapy for radiation myelopathy. The present work may provide preclinical data for the treatment of radiation myelopathy using human MSCs.
